# Myocardial Ischemia, a Rare Presentation of Meckel’s Diverticulum

**DOI:** 10.51894/001c.12844

**Published:** 2020-06-08

**Authors:** Trevor A Nessel, Connor C. Kerndt, Zaid J. Shareef, Christopher Doig

**Affiliations:** 1 OMS-IV Michigan State University College of Osteopathic Medicine; 2 Spectrum Health/Michigan State University College of Human Medicine; 3 Internal Medicine Garden City

**Keywords:** gastrointestinal bleed, nstemi, myocardial ischemia, angina, congenital anomaly, meckel’s diverticulum

## Abstract

**CONTEXT:**

Meckel’s diverticulum is a rare congenital anomaly of the gastrointestinal tract. It is typically asymptomatic and found incidentally in the work-up of another medical complaint. However, it has been known to cause complications in a minority of cases.

**METHODS:**

This case involves an elderly male in his early 80’s who presented to the emergency department with a 2-day history of emesis and hematochezia, in addition to sudden onset syncope and angina-like symptoms. Serial electrocardiograms demonstrated diffuse ST-segment depressions, consistent with myocardial ischemia. The patient underwent laboratory testing, imaging, endoscopy, and a subsequent exploratory laparotomy.

**RESULTS:**

Laboratory results revealed lactic acidosis, anemia, and leukocytosis. Upper endoscopy resulted in negative findings. Imaging, including CT-scan and Technetium-99 RBC scan, visualized a gastrointestinal bleed. However, the arterial embolization procedure was unable to stop the bleeding diverticulum. Exploratory laparotomy revealed an infarcted Meckel’s diverticulum.

**CONCLUSIONS:**

This case demonstrates the importance of clinicians generating a wide differential when evaluating a gastrointestinal bleed, and considering Meckel’s diverticulum as a potential cause of a bleed with an unknown source. The primary test to diagnose a Meckel’s diverticulum is a Technetium-99 RBC scan. However, visualization via exploratory laparotomy is the best test for definitive diagnosis. The decision to intervene surgically earlier can limit mortality with symptomatic Meckel’s diverticula.

## INTRODUCTION

Meckel’s diverticulum (MD) represents the most common congenital defect of the gastrointestinal (GI) system. MD originates from the omphalomesenteric duct, which functions to connect fetal intestines to the yolk-sac during embryogenesis, and may remain a true diverticulum if it fails to regress.^1^ In a majority of patients affected, the remnant MD generally fails to induce symptoms or complications. The classic “rule of 2s” summarizes several defining features of MD: two feet proximal to the ileocecal valve, two inches in length, 2% of the population, and two different ectopic tissues (gastric and pancreatic).^2^

Studies predict that only 6% of people with MD present with symptomatic complications during their lifetime.^3^ For the majority of patients, a diagnosis of MD is established while undergoing diagnostic testing for other medical problems and is detected incidentally.^2^ There are several dangerous complications that can manifest in the adult population, including bowel obstruction, intussusception (telescoping of bowel), diverticulitis, perforation, and hemorrhage.^3^ Rate of complications and age typically display an inverse relationship, as complications manifest in 4% of patients under 20 years old, 2% of patients under 40 years old, and nearly 0% of patients older than 40 years old.^4^

Common etiologies of a lower GI bleed include diverticulosis, hemorrhoids, ischemia.^5^ However, MD should not be overlooked as a potential cause of a GI bleed. This case represents an atypical presentation of MD manifesting as a Non-ST-Elevation Myocardial Infarction (NSTEMI) in a geriatric patient.

## CASE REPORT

A male in his early 80’s presented to the emergency department with a two-day history of emesis, hematochezia, and subsequent syncope. Prior to his syncopal episode, he endorsed brisk diaphoresis. At the time of presentation, the patient denied angina or recent trauma.

The patient’s past medical history included a myocardial infarction, coronary artery disease, dyslipidemia, gastroesophageal reflux disease, and duodenal ulcer. His past surgeries included a cholecystectomy, coronary artery bypass grafting, and three percutaneous coronary interventions with placement of three stents. Two months before presentation the patient underwent percutaneous coronary intervention for drug-eluting stenting of an unknown vessel. His social history was significant for a 20-pack-year smoking history, moderate alcohol consumption, and no illicit drug use.

Evaluation of the patient’s presenting vitals revealed a BP 116/59 mmHg and heart rate of 92 bpm. Laboratory testing suggested anemia with a hemoglobin of 6.7 g/dL, in addition to leukocytosis and lactic acidosis (Table 1). Physical examination was significant for dark clotted blood within the rectal vault. Initial electrocardiogram demonstrated ST-depressions in leads II, V2, V4, and V5, along with concurrent troponin elevation, signifying signs of initial ischemia (see blue arrows in Figure 1A).

Twenty minutes later, while still in the emergency department the patient began to endorse substantial substernal angina. The ensuing electrocardiogram exhibited worsening diffuse ST-segment depressions from previous testing, suggestive of severe global ischemia (Figure 1B). After treatment with nitroglycerin and an initial blood transfusion, the patient was stabilized and transferred to the intensive care unit for escalation of care.

**Table 1:** Initial Emergency Room Laboratory Values

**Labs Ordered**	**Patient Value**	**Reference Range**
White Blood Cells	16.3	4.8-10.8 K/cumm
Hemoglobin	6.7	13.9-16.3 g/dL
Hematocrit	21.4	39.0-55.0 %
Red Blood Cells	2.3	4.30-5.90 M/uL
Platelets	214	130-375 K/uL
Lactic acid (v)	3.8	0.4-2.0 mmol/L
Troponin	0.36	< 0.04 ng/mL
Fecal Occult Blood Test (FOBT)	Positive	-

**Figure 1A:**
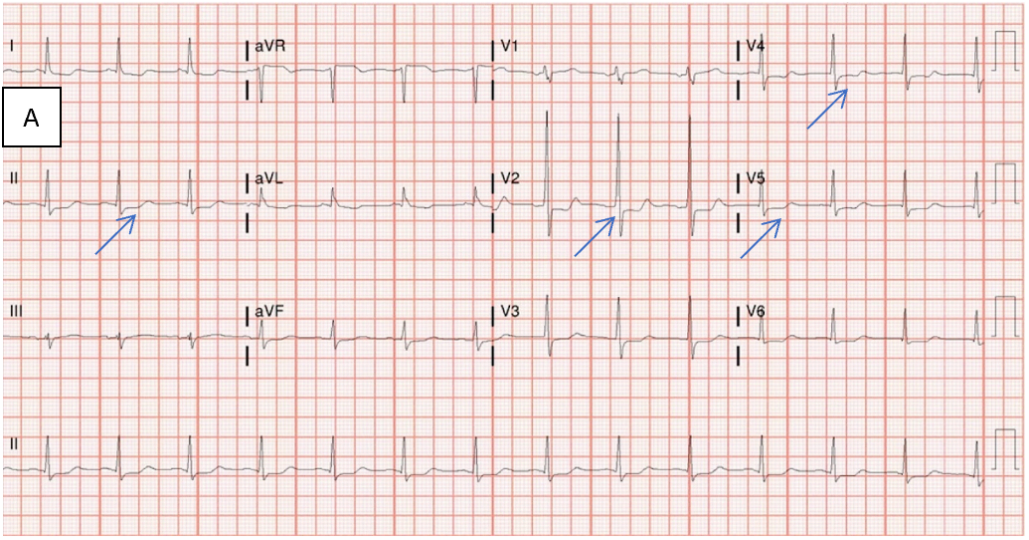
Electrocardiogram on Day 1 of hospital admission before chest pain. Findings: Sinus rhythm; probable posterior infarct with ST-depressions in leads II, V2, V4, V5 (blue arrows).

**Figure 1B:**
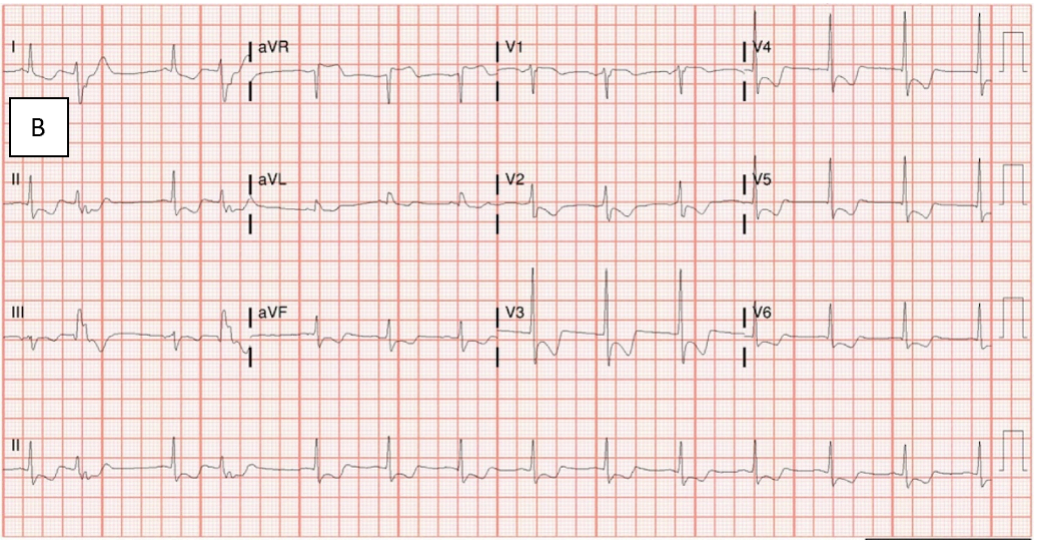
Electrocardiogram after the onset of initial chest pain. Findings: Sinus rhythm; Diffuse ST-depression with ST-elevation in aVF, consistent with severe global ischemia; Short PR intervals are now present; Prolonged QT intervals are now present.

A CT-scan of the abdomen initially revealed diffuse diverticulosis without evidence of an acute gastrointestinal bleed. An emergent esophagogastroduodenoscopy (EGD) followed, which failed to identify the source of bleeding. The following day, a CT-scan with contrast assessed for intra-abdominal causes of the ongoing acute blood loss anemia. Active diverticular bleeding near the hepatic flexure was noted on CT-scan and was confirmed with focal uptake on a Technetium-99 RBC scan. Arterial embolization was performed at this site but was unsuccessful in terminating the bleed.

On hospital Day 3, the patient experienced another episode of angina which resolved with the application of sublingual nitroglycerin. Subsequent electrocardiography demonstrated worsening ST-segment changes and new premature ventricular contractions in comparison with the previous electrocardiogram (Figure 1C).

**Figure 1C:**
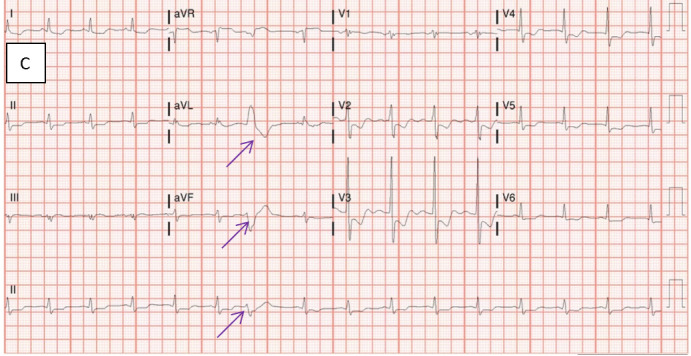
Electrocardiogram prior to exploratory laparotomy. Findings: Worsening ST-depression with ST-elevation consistent with severe global ischemia (left main or multivessel disease); Premature ventricular complexes are present; Prolonged QT intervals are present; Possible ischemia is still present; Premature ventricular complexes are now present (purple arrows).

**Figure 2:**
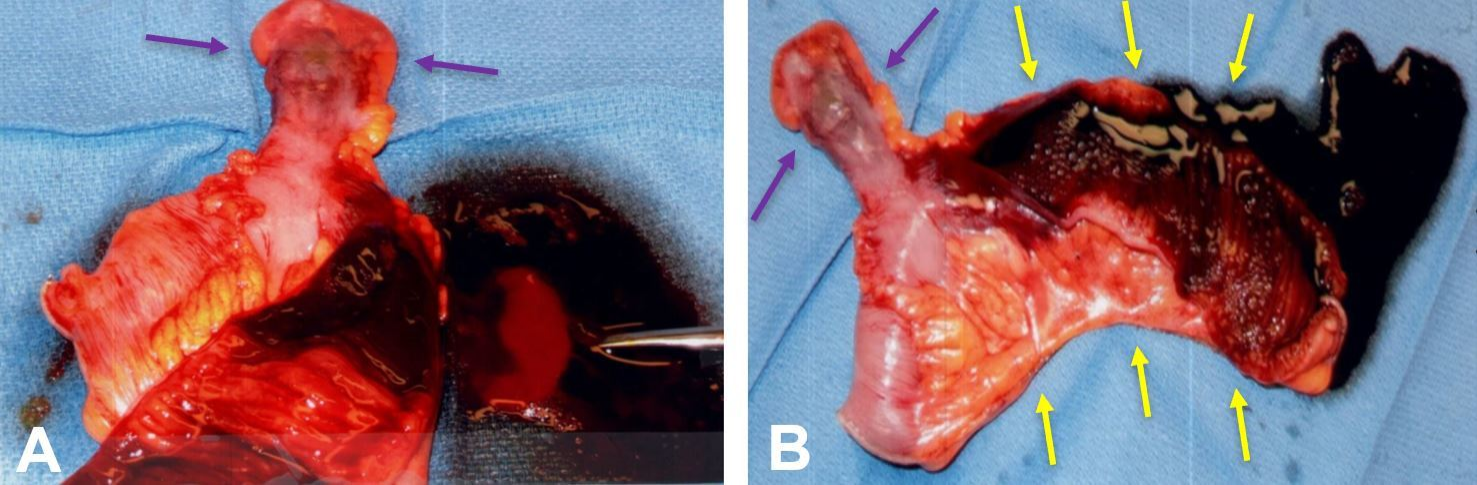
Gross specimen consisting of the infarcted Meckel’s diverticulum and portions of ileum. shows the Meckel’s diverticulum (purple arrows), while (B) shows the Meckel’s diverticulum (purple arrows) and a segment of ileum (yellow arrows). Mild acute inflammation is present within the mucosa. The Meckel’s diverticulum, located adjacent to the purple dusky area, measures 4.5 cm in length and 1.5-2.3 cm in width. The ileum measures 17.0 cm in length, with a circumference ranging from 4.0 to 5.0 cm.

Despite arterial embolization, the patient remained anemic with leukocytosis and thrombocytopenia (Table 2). The patient’s hemodynamic stability deteriorated despite receiving numerous blood transfusions, and thus he underwent an emergent exploratory laparotomy for definitive identification. Surgical intervention unsheathed a bleeding, infarcted MD with concurrent infarcted ileum. Both the infarcted ileum and MD were surgically resected on Day 3, resulting in bleeding cessation (Figure 2).

Post-procedure, the patient’s hemoglobin progressively increased, attaining a more normal hemoglobin level of 12.2 g/dL. The patient’s hematochezia gradually tapered to a halt by Postop Day 6. Over the course of the 10 day hospital admission, he received a total of 22 units of packed red blood cells, 11 units of fresh frozen plasma and 4 units of platelets. The patient was transferred to inpatient hospital rehabilitation on Day 10 after successful stabilization.

**Table 2:** Pre-exploratory laparotomy Laboratory Values Day 3

**Labs Ordered**	**Patient Value**	**Reference Range**
White Blood Cells	11.9	4.8-10.8 K/cumm
Hemoglobin	8.5	13.9-16.3 g/dL
Hematocrit	25.7	39.0-55.0 %
Red Blood Cells	2.91	4.30-5.90 M/uL
Platelets	84	130-375 K/uL
Lactic acid (v)	0.9	0.4-2.0 mmol/L
Troponin	4.05	< 0.04 ng/mL
Fecal Occult Blood Test (FOBT)	N/A	-

## DISCUSSION

MD was first described anatomically and embryologically by Johan Friedrich Meckel in 1809.^6^ In utero, the omphalomesenteric duct connects the embryonic midgut to the yolk sac anteriorly, allowing the transportation of nutrients to the midgut.^1^ Between the seventh and eighth week of gestation, the omphalomesenteric duct typically narrows and involutes as it no longer plays a functional role in development.^7^ In MD, the proximal omphalomesenteric duct fails to involute which leads to an outpouching of the small intestine.

MD is a rare GI congenital anomaly that occurs in 2% of the population. Of those with MD, very few are symptomatic and even fewer present with myocardial ischemia secondary to a GI bleed. Only 4-6% of MD patients will become symptomatic during their lifetime.^3^ Males and females are equally affected by MD, but symptomatic MD preferentially inflicts males at a ratio of 4:1.^8^ It has been hypothesized that males may be more commonly symptomatic due to higher innate gastrin levels leading to higher levels of acid secretion by their ectopic gastric mucosa.^9^

Most children with MD remain asymptomatic, however, symptomatic MDs are more common in children than adults.^10^ Children who experience complications present most commonly with intestinal obstruction, intussusception, and volvulus (twisting of bowel).^11^ In adults, the most common symptomatic presentation is intestinal obstruction, followed by enteroliths (gastrointestinal calculi), torsion, bowel ischemia/necrosis, bleeding, and diverticulitis.^12^

Although geriatric presentations like this are uncommon, a majority of those patients who are symptomatic present with diverticulitis.^7^ Malignancies can rarely originate from the site of MD. Of MD complications, oncogenic processes account for 0.5-4.9% of symptomatic presentations, most commonly attributed to sarcomas.^13^ Carcinoid tumors and adenocarcinomas have also demonstrated the capability to originate from MD.^13^

This case presentation was unusual due to the patient’s age, symptomatic nature, and cardiac manifestations. Few cases of MD have illustrated symptomatic manifestations in patients greater than 80 years of age. The majority of symptomatic patients present in childhood, and most of those who present as an adult do so in early-mid adult life. By 76 years of age, the risk of MD complications has been estimated to be nearly 0%.^14^ Furthermore, this presentation demonstrates an exceptional finding given the symptomatic presentation with cardiac manifestations of an NSTEMI. The authors review of the literature in this area yielded only one previously documented case of MD manifesting as angina secondary to myocardial ischemia.^7^

Given this atypical presentation, the mechanism which caused this man’s MD is important to delineate. The most likely scenario suggests that his MD served as a site for bowel torsion, which led to compression of his adjacent colonic vasculature. Impaired blood flow caused tissue ischemia, necrosis, and subsequent bleeding. His acute blood loss anemia secondary to tissue infarction required an increase in his cardiac index to maintain adequate perfusion to other vital organs. The increased need for cardiac output induced significant stress on the myocardium. This demand led to acute angina with concomitant ST-segment depression.

Diagnosis and treatment of MD remains challenging due to the lack of definitive imaging techniques and diagnostic procedures. This condition is often first noticed as a possible anomaly on CT-scan, which generally shows a MD outpouching with tubular collections of air and enhanced wall thickening.^15^ Although MD may be first discerned on CT-scan, a Technetium-99 RBC scan is the diagnostic test of choice to identify a MD.^16^ A Technetium-99 RBC scan uses 99-technetium pertechnetate, a radioactive tracer, to visualize the ectopic gastric mucosa-containing MD.^17^ Approximately 50% of MD contain gastric mucosa which allows for prompt identification.^17^

A Technetium-99 RBC scan is exceptionally helpful in cases of bleeding MD as gastric mucosa is known to carry a significant risk of bleeding.^17^ Technetium scans will fail to identify some cases of MD because not all cases contain gastric remnants. The specificity and sensitivity of the Technetium-99 RBC scan is 85% and 95% respectively in children and 60% and 9% respectively in adults.^18^ These statistics elucidate the greater difficulty of establishing a diagnosis of MD in the adult population. The reduced sensitivity in adults is partly due to the reduced likelihood of an adult MD to express ectopic gastric mucosa.^7^ Thus, MD cannot be ruled out based solely on negative nuclear image findings. The only definitive method to diagnose a bleeding MD is with an exploratory laparotomy.

## CONCLUSIONS

The clinical presentation of myocardial ischemia in the presence of an infarcted MD in an elderly patient is an extremely unusual finding. There are very few case reports that have described symptomatic adult MD presentations. This case illustrates that not only can MD become symptomatic, but it can potentiate life-threatening consequences as well. It is important to consider atypical presentations such as angina. The presence of a GI bleed secondary to a MD can place stress on vital organs such as the heart, particularly in older cardiac compromised patients.

Testing to diagnose MD, especially in the adult population, remains a challenge due to the relatively lower sensitivity and specificity of available diagnostic tests. The only way to definitively diagnose a GI bleed is through direct visualization via endoscopy or exploratory surgical laparotomy. It is therefore essential to generate a wide differential diagnosis when evaluating a GI bleed. MD should be considered a possible cause of a GI bleed with an unidentifiable source. This case demonstrates how early identification can serve to prevent unnecessary blood transfusions, hemodynamic instability, and mortality.

### Conflict of Interest

The authors declare no conflict of interest.
